# Flora of herbaceous and arboreous plants in Komaba Campus of the University of Tokyo, Japan

**DOI:** 10.3897/BDJ.9.e73177

**Published:** 2021-09-27

**Authors:** Seikan Kurata, Naoko Ishikawa, Diego T. Vasques, Masayuki U. Saito, Osamu Kurashima, Motomi Ito

**Affiliations:** 1 Department of General Systems Studies, Graduate School of Arts and Sciences, University of Tokyo, Meguro, Tokyo, Japan Department of General Systems Studies, Graduate School of Arts and Sciences, University of Tokyo Meguro, Tokyo Japan; 2 Botanical Gardens, Osaka City University, Katano, Osaka, Japan Botanical Gardens, Osaka City University Katano, Osaka Japan; 3 Center for Global Communication Strategies, College of Arts and Sciences, University of Tokyo, Meguro, Tokyo, Japan Center for Global Communication Strategies, College of Arts and Sciences, University of Tokyo Meguro, Tokyo Japan; 4 Department of Food, Life and Environmental Sciences, Faculty of Agriculture, Yamagata University, Tsuruoka, Yamagata, Japan Department of Food, Life and Environmental Sciences, Faculty of Agriculture, Yamagata University Tsuruoka, Yamagata Japan; 5 National Museum of Nature and Science, Taito, Tokyo, Japan National Museum of Nature and Science Taito, Tokyo Japan

**Keywords:** arboreous plants, herbaceous plants, plant survey, urban area

## Abstract

**Background:**

Recent studies revealed that green spaces in urban areas are critical for conservation of native biodiversity and that assessment of the present flora of green spaces in urban areas is critical for protection of the native biodiversity. The Komaba Campus of the University of Tokyo embraces a relevant green area, located in a highly urbanised area in Tokyo Metropolis (35.66 N 139.68 E, Japan). The total area of this Campus is 25.4 ha, from which, 4.5 ha are covered by vegetation. Although intense urbanisation can be observed around the Campus, new insect species had been reported for the Campus area, suggesting that the biodiversity on the Campus still demands some attention. Differently from fauna surveys, no flora survey has been done for more than 30 years on the Campus. In this study, we have extensively surveyed the plants diversity on the Komaba Campus of the University of Tokyo, aiming for an update of the plants list on this green urban area in Tokyo.

**New information:**

The survey covered all herbaceous and arboreous plants growing wild on the Campus. Garden plants were excluded in this survey because these plants were supposed to be cultivated. The final dataset contained, in total, 324 taxa, from which 234 were herbaceous plants and 90 were arboreous plants. The top three taxa are as follows: Poaceae (38 taxa), Asteraceae (34 taxa) and Rosaceae (14 taxa), respectively. This is the first update to the Flora of the Komaba Campus of the University of Tokyo in 30 years and represent an important contribution to conservation of native species in the Tokyo metropolis.

## Introduction

Assuming a direct correlation between urbanisation and development, urban development can lead to biotic homogenisation for native plant species (Kühn and Klotz 2006). One hypothesis says that, although biotic homogenisation occurs only in very urbanised landscapes, this homogenisation originates from non-native invasion without local native extirpation (Blouin et al. 2019). Particularly, the occurrence of non-native plant species tends to increase along an urban-rural gradient, with a biased distribution towards the centre of urban areas (Whitney 1985, Kowarik 1995, McKinney 2002). In this scenario, native plant species survive in urban areas without being driven away by non-native plant species, co-existing there. Alternatively, an opposing hypothesis suggests that invasions of non-native species causes native species to be exterminated overtime (Fritts and Rodda 1998, Wilcove et al. 1998). Thus, assessments of the present flora and fauna of green spaces in urban areas are critical for protecting native biodiversity.

People living in cities create green spaces (e.g. parks, pavement, gardens and lawns, road and railroad verges, vacant lots and roofs) as artificial habitats (Williams et al. 2009) and a recent study revealed that green spaces in urban areas are critical for conservation of native biodiversity and threatened species (Ives et al. 2015). The Komaba Campus of the University of Tokyo (25.4 ha) is located in a highly urbanised area in Tokyo Metropolis (Japan) and the Campus has a low area coverage of green spaces (4.5 ha). Although urbanisation has proceeded around the Campus, some new insect species had been recorded in this Campus in recent years (Yasunaga et al. 2013, Ishikawa et al. 2015), leading us to suppose a relatively high biodiversity is maintained on the Campus. Some surveys of fauna (Cerambycidae and Pentatomoidea) were performed on the Komaba Campus of the University of Tokyo in recent years (Ishikawa et al. 2015, Kishimoto-Yamada et al. 2017), but the only flora survey available for the area was performed 36 years ago (Meguro Ward office 1985). Thus, an updated study, including correct identification and classification of the plants found on Campus, is made necessary. It is expected that this flora information will provide the basic data for understanding the importance of green spaces in an urban area in Tokyo, Japan.

## Sampling methods

### Study extent

All specimens were collected in the Komaba I Campus of University of Tokyo, Komaba, Meguro City, Tokyo, Japan. The total area of the Campus is 25.4 ha, from which 20.9 ha consist of buildings and several athletic fields (http://www.c.u-tokyo.ac.jp/info/about/facts/lands/index.html, accessed on June 2021). The remaining 4.5 ha are covered by vegetation, being used as our investigation site. Annual temperature in Tokyo varies between 5.2°C and 26.4°C (January to August annual average from 1981 to 2010), with mean annual precipitation of 1,598.2 mm (Japan Meteorogical Agency, https://www.data.jma.go.jp/obd/stats/etrn/view/nml_sfc_ym.php?prec_no=44&block_no=47662, accessed in June 2021).

### Sampling description

Plants with reproductive structures were collected between April 2017 and May 2019 at different times of the year. Collection was interrupted between December and February, when few plants with reproductive structures are observed. Sampling was exhaustive and performed once a month during daytime, virtually covering all vegetation areas on the Campus. Two individuals were collected for each taxon, one of those being preserved and registered at the Komaba Museum, University of Tokyo, Meguro City, Japan (KMUT). The whole plant body was collected (including roots), with the exception of large individuals (e.g. *Cirsiumvulgare* (Savi) Ten. and *Alcearosea* L.), from which only shoots were collected. For fern specimens, rhizomes and fertile leaves were collected to allow identification. Collected samples were immediately mounted as vouchers and preserved at room temperature using humidity-absorbing sheets. Vouchers’ deposit numbers were issued by KMUT.

Plant species were identified according to The Handbook of Common Grasses (Koba et al. 2012), The Handbook of Sedges in Japan (Katsuyama and Kitagawa 2014), The Handbook of Ferns and Fern Allies (Kitagawa 2009), Wild Flowers of Japan, Plains, Seaside and Hills (Hayashi et al. 2017), Wild Flowers of Japan, Mountainside (Kadota et al. 2017), The Standard of Ferns and Lycophytes in Japan 1, 2 (Ebihara 2016a, Ebihara 2016b) and Leaves of Trees (Hayashi 2018). The classification system follows APG IV (The Angiosperm Phylogeny Group et al. 2016) and family, genus and Japanese names were confirmed by Ylist (Yonekura and Kajita 2003).

## Geographic coverage

### Description

**Figure 1.**
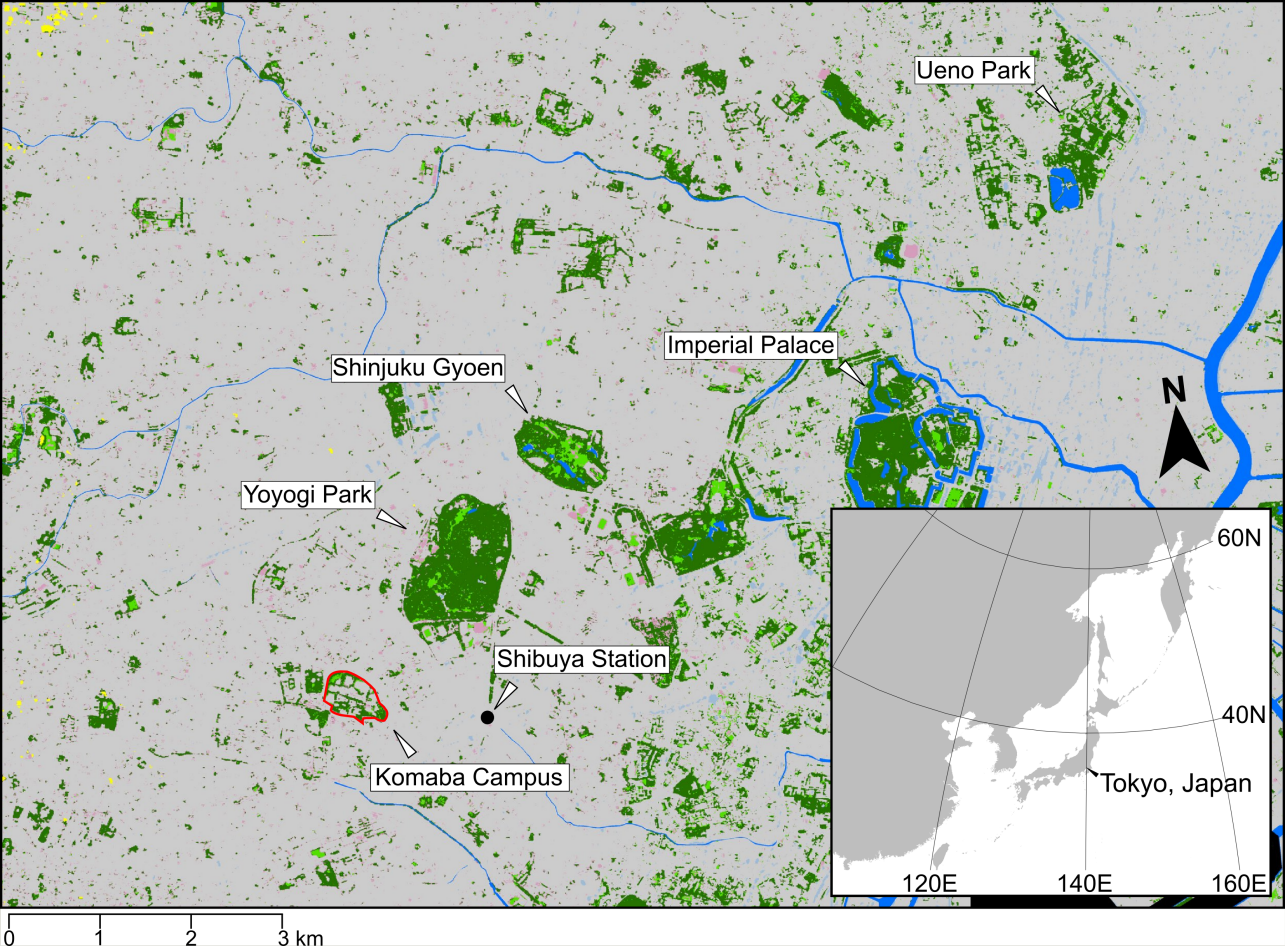
Survey area (highlighted in red) and the adjacent green spaces of Tokyo, such as the Yoyogi Park, Shinjuku Gyoen, the Imperial Palace and Ueno Park.

The survey was performed at the Komaba I Campus of University of Tokyo, Komaba, Meguro City, Tokyo, Japan. The Campus is in a residential area, adjacent to the huge business district of Shibuya and relatively close by other green spaces in Tokyo, such as the Yoyogi Park, Shinjuku Gyoen, the Imperial Palace and Ueno Park (Fig. 1).

### Coordinates

35.658 and 35.664 Latitude; 139.681 and 139.689 Longitude.

## Taxonomic coverage

### Description

**Figure 2.**
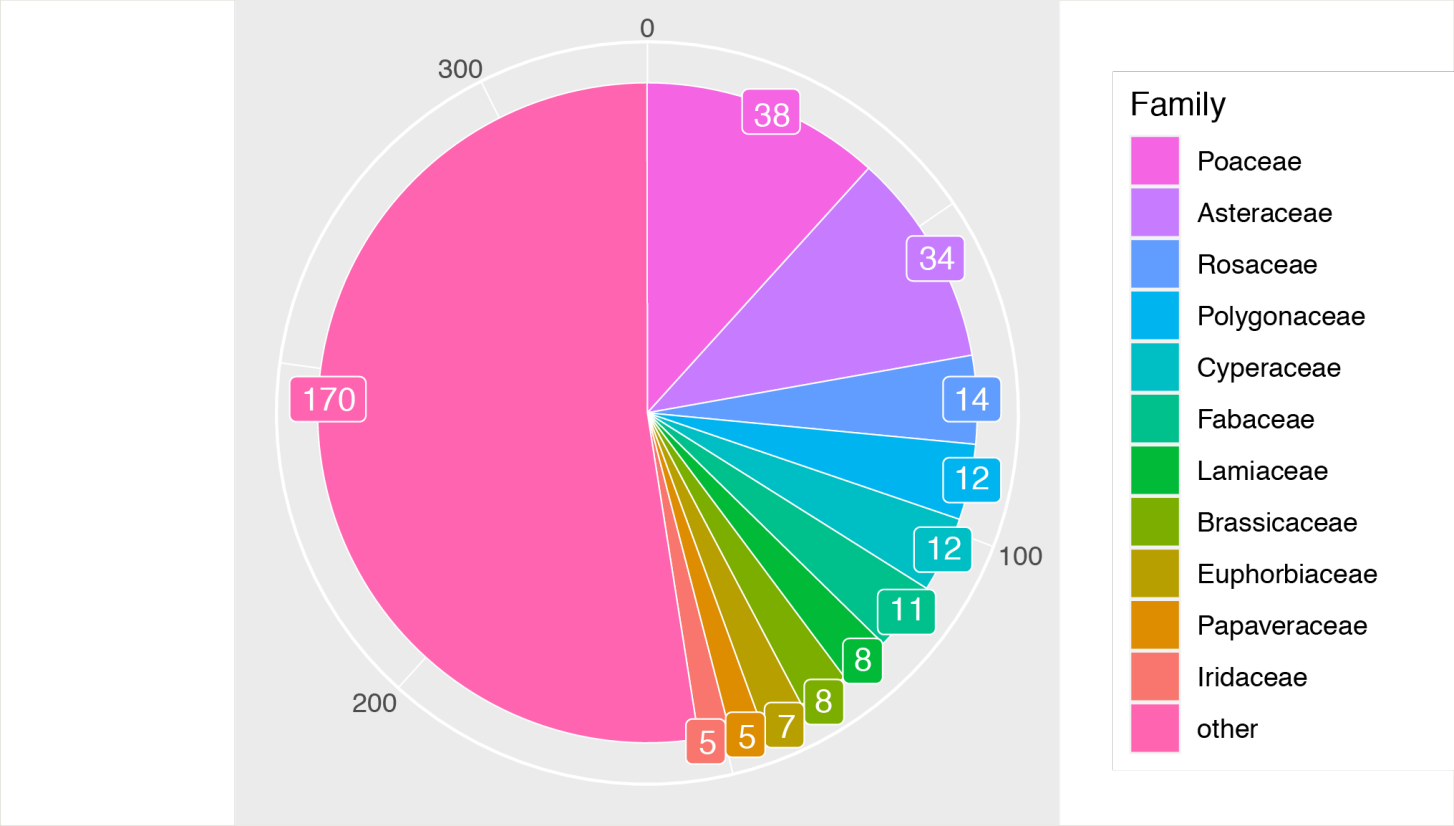
Pie chart describing the diversity of taxonomic families found on the Flora survey on Komaba Campus of the Univeristy of Tokyo. Families were identified under APG IV (2016). Numbers on the chart indicate the number of taxa identified under each family. In total, 99 different family taxa were identified in this survey.

The survey covers all herbaceous plants which grow wild in the Campus and arboreous plants which grow wild or are cultivated. Garden plants were excluded in this survey because these plants were supposed to be cultivated. The dataset contains in total 324 taxa: 234 herbaceous plants and 90 arboreous plants (Suppl. material 1, Kurata and Vasques 2021). The most common plant families found on the Campus were Poaceae (n = 38 taxa, 11.7%), Asteraceae (n = 34 taxa, 10.5%) and Rosaceae (n = 14 taxa, 4.3%), from a total of 99 families identified (Fig. 2).

## Temporal coverage

### Notes

Date range: April 2017 – May 2019.

## Usage licence

### Usage licence

Creative Commons Public Domain Waiver (CC-Zero)

## Data resources

### Data package title

Herbaceous and arboreous plants list on the Komaba Campus of University of Tokyo, Japan

### Resource link

https://www.gbif.org/dataset/63b42483-48e8-4dd8-acca-a8ad35e72f45

### Number of data sets

1

### Data set 1.

#### Data set name

Komaba_Flora

#### Number of columns

32

#### 

**Data set 1.** 

Column label	Column description
parentNameUsage	Genus name
occurrenceID	Unique occurrence ID
modified	The most recent date-time on which the resource was changed
language	Thelanguage of the resource
basisOfRecord	Type of the records
acceptedNameUsage	Same as “scientificName”
scientificName	Scientific name for the species
kingdom	Taxonomical kingdom
class	Taxonomical class
order	Taxonomical order
family	Taxonomical family
genus	Taxonomical genus
specificEpithet	Specific epithet for the species
infraspecificEpithet	Infraspecific ranks
identificationRemarks	Comments or notes about the identification
taxonRank	Most specific identified rank for the taxon
scientificNameAuthorship	Author name for the species
taxonomicStatus	Status for scientific name usage
recordedBy	Collector name for the specimens
country	Country of collection
stateProvince	Province of collection
municipality	City of collection
locality	Other location data
minimumElevationInMeters	Minimum elevation (metres)
maximumElevationInMeters	Maximum elevation (metres)
decimalLatitude	Decimal latitude
decimalLongitude	Decimal longitude
identifiedBy	Determination authors
recordNumber	Specimen number
license	Creative Commons licence
eventDate	The verbatim original representation of the date and time information for an Event
coordinateUncertaintyInMeters	The horizontal distance from the given decimalLatitude and decimalLongitude in metres

## Supplementary Material

F2C3C145-C1FD-5918-91B4-E74C7BD9C0DF 10.3897/BDJ.9.e73177.suppl1 Supplementary material 1 The list of samples collected in Komaba I Campus of University of Tokyo Data type Occurrences Brief description List of herbaceous and arboreous plants list in the Komaba Campus of the University of Tokyo, recorded in 2017 – 2019. File: oo_578944.csv https://binary.pensoft.net/file/578944 Seikan Kurata, Naoko Ishikawa, Diego Tavares Vasques, Masayuki U Saito, Osamu Kurashima, Motomi Ito
